# Approach Towards the Development of Digital Twin for  Structural Health Monitoring of Civil Infrastructure: A Comprehensive Review

**DOI:** 10.3390/s25010059

**Published:** 2024-12-25

**Authors:** Zhiyan Sun, Sanduni Jayasinghe, Amir Sidiq, Farham Shahrivar, Mojtaba Mahmoodian, Sujeeva Setunge

**Affiliations:** School of Engineering, RMIT University, 124 La Trobe Street, Melbourne, VIC 3000, Australia; s3950682@student.rmit.edu.au (Z.S.); s3751824@student.rmit.edu.au (S.J.); amir.sidiq@rmit.edu.au (A.S.); s3737042@student.rmit.edu.au (F.S.); sujeeva.setunge@rmit.edu.au (S.S.)

**Keywords:** digital twin, civil infrastructure, structural health monitoring, virtual model, asset management

## Abstract

Civil infrastructure assets’ contribution to countries’ economic growth is significantly increasing due to the rapid population growth and demands for public services. These civil infrastructures, including roads, bridges, railways, tunnels, dams, residential complexes, and commercial buildings, experience significant deterioration from the surrounding harsh environment. Traditional methods of visual inspection and non-destructive tests are generally undertaken to monitor and evaluate the structural health of the infrastructure. However, these methods lack reliability due to the need for instrumentation calibration and reliance on subjective visual judgments. Digital twin (DT) technology digitally replicates existing infrastructure, offering significant potential for real-time intelligent monitoring and assessment of structural health. This study reviews the existing applications of DTs across various sectors. It proposes an approach for developing DT applications in civil infrastructure, including using the Internet of Things, data acquisition, and modelling, together with the platform requirements and challenges that may be confronted during DT development. This comprehensive review is a state-of-the-art review of advancements and challenges in DT technology for intelligent monitoring and maintenance of civil infrastructure.

## 1. Introduction

Well-developed and maintained civil infrastructures, including buildings, pipelines, roads, bridges, tunnels, and other assets, significantly contribute to economic growth and social well-being for developed and developing countries [1]. In 2023, the real Gross Domestic Product (GDP) growth rate for several major developed countries was between 0.5% and 4.6%, including 2.7% in the USA, 0.5% in the United Kingdom, 0.9% in Japan, 4.6% in China, and 1.5% in Australia. In many developed countries, civil infrastructures contribute 6% to 12% of the GDP [2]. In Australia, in 2023, 9.2% of the GDP growth was attributed to the monitoring and maintenance of major civil infrastructures (i.e., 9.2% of 1.5%), including 877,651 km of roads, 33,004 km of open railways, 53,000 bridges, 17 nationally significant ports, 571 dams and 613 airports [3]. Regarding the energy infrastructure, Australia has one of the world’s largest energy generation, storage, and distribution networks, including 40,000 km of high-voltage transmission lines, 770,000 km of lower-voltage distribution lines and 1500 km of interconnectors [4]. Population growth and increased infrastructure usage demands, coupled with harsh environmental conditions, impose significant stress on these structures, affecting their serviceability, performance, and structural integrity [5]. Additionally, ageing civil infrastructure assets are prone to structural failure if maintenance is insufficient [6]. It is crucial to monitor infrastructure assets and promptly address any potential damage closely.

In structural analysis, damage refers to changes that negatively impact the performance of civil infrastructure, including any changes to the boundary conditions, system connectivity, and material and geometric properties [7]. Conventionally, structural damage is observed by undertaking routine visual inspections and non-destructive testing (NDT). Based on qualitative approaches, visual inspections have a high degree of inconsistency due to different factors, including (1) inspector subjectivity, (2) site accessibility, and (3) inspection result verification [8,9,10,11]. However, standard NDT methods undertaken for asset inspection are usually time-consuming, require frequent calibration and are limited to defect detection for specific types at a local scale. For instance, the X-radiography is limited to substrates with low absorption [12]. In response to the continuous rising demand for more efficient, systematically consistent infrastructure integrity assurance conditions, structural health monitoring (SHM) has been proposed and widely implemented worldwide in civil infrastructure [13]. Its original intention is to provide continuous diagnosis of the constituent materials of the asset [14]. However, due to recent developments in computer science, data science, electronics, mechanics, material science, and information technology, data richness, volume, and accuracy have been vastly improved overall. This development facilitates the growth of modelling and simulation technology and gradually exhibits a more substantial capability to model the physical world with higher fidelity.

The authors comprehensively reviewed the literature on digitalised approaches for maintaining civil infrastructure, emphasising their potential to surpass traditional inspection methods in accuracy and effectiveness. A DT, a virtual replica of the physical world, is an emerging concept in civil engineering and has shown remarkable promise in enabling real-time structural performance evaluation. This review provides an in-depth analysis of the background and advancements in DT development for civil infrastructure. It explores methods for model creation using data-driven approaches, the integration of IoT technologies and connectivity, platforms for ensuring reliability and data interpretation, and the challenges that may arise in the evolution and implementation of DTs within the civil engineering domain.

## 2. Background of DT Development

**Figure 1:**
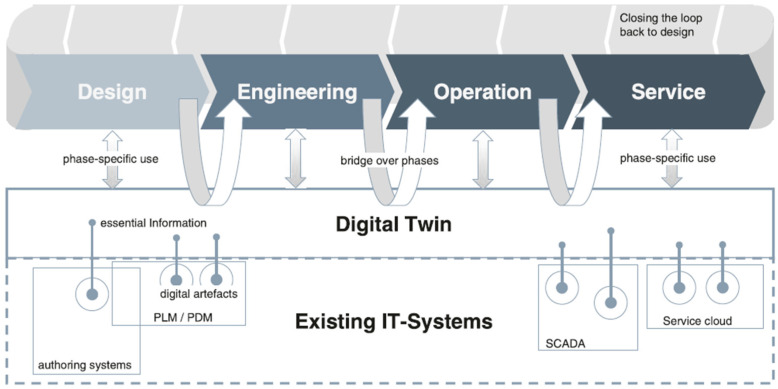
DT uses generated data during the asset life cycle to inform subsequent phases and future developments [11].

In recent years, the burgeoning advancement of Internet of Things (IoT) technologies has precipitated a notable surge in interest surrounding the concept of a DT. The DT concept was initially undertaken by Grieves and Vickers [15] for product life cycle management and stated that in product life cycle management, a DT is a mirror image of a physical process that is articulated alongside the process in question, usually matching precisely the operation of the physical process which takes place in real-time. The definition of DT has evolved across various industries. Table 1 lists DT definitions from the literature review, primarily in manufacturing and aircraft development. DT is proposed to federate all essential asset-related information and to present it in an interactivity-fostering manner. Asset owners and managers benefit from accurate data acquisition, consistent project delivery, and efficient data reuse, as shown in Figure 1. DT can also embrace and utilise modern technological instrumentation and methods such as sensor networks, data communication, and simulation technologies [16]. Furthermore, the abundance of collected data, in conjunction with advancements in data processing and interpreting techniques, allows for the development of data modelling and simulation approaches to visualise a physical asset’s static and dynamic status in real time [17]. Compelled by the technological push (e.g., IoT, virtual reality) and the demand-pull (e.g., remote inspections, model behaviour predictions via algorithms), the DT development represents a significant shift in technology, with its market projected to reach AUD 20.5 billion by 2025, reflecting a compound annual growth rate of 37.87% [18].

Compared to conventional surveillance systems, DT offers a broader range of sensing capabilities with improved real-time responsiveness. They also incorporate advanced data analysis capabilities and more user-friendly interfaces. Consequently, DTs enhance our understanding and predictive capabilities regarding machine and system performance. They also facilitate the optimisation of business operations for consumers and asset owners.

**Figure 2:**
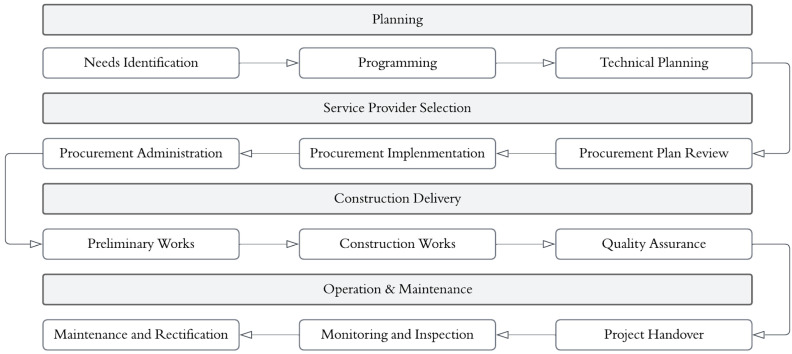
Overview of the current infrastructure project life cycle.

The status of an infrastructure project life cycle (Figure 2) involves gathering information generated by different experts/parties using different software, which is often not interchangeable and potentially leads to different judgements. Therefore, cross-disciplinary teamwork is essential in the early stages of the design process to achieve a successful integration of assets, community, and natural and economic systems [29].

Conventional infrastructure projects continuously suffer from suboptimal construction delivery speed. Potential causes include contradictory design inputs from standalone specialists and late approval communications from supervising officials. Another related issue is obtaining fundamental data, such as asset conditions levels or maintenance details, which are generally unavailable. The above-mentioned issues restrict processing levels and introduce errors, therefore generating erroneous decisions on asset lifecycle management. It also limits the potential for establishing asset maintenance regimes through benchmarking maintenance practises by asset performance before and after maintenance [30]. Literature in infrastructure engineering highlights the deficiencies in inspection procedures, defect identification, and asset maintenance [31]. DT technology has been proven capable of processing, evaluating, and integrating real-time monitoring data and, consequently, accurate SHM of infrastructure assets [32]. Another beneficial aspect of DT is facilitated collaboration, which allows for faster and more transparent communication between the designers and clients within the planning stage of the life cycle of an infrastructure by using real-time mapping of the physical product [33].

**Figure 3:**
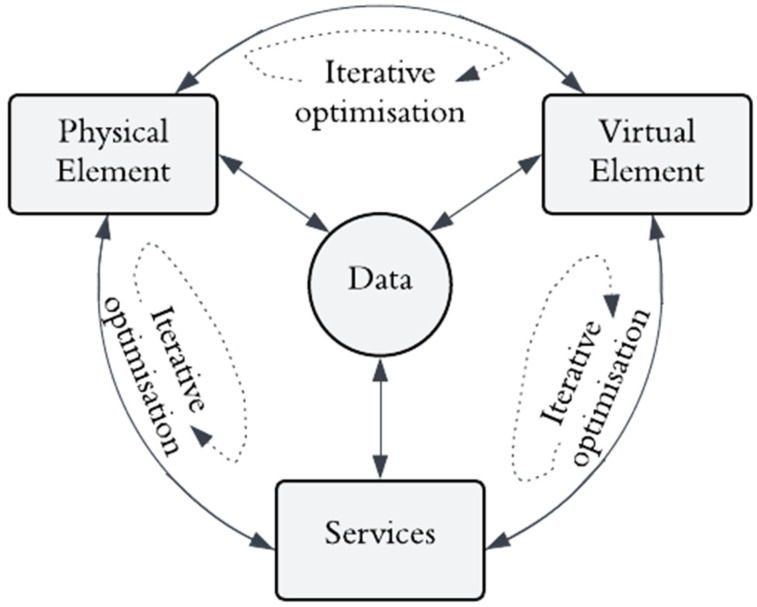
Components of a DT.

In civil engineering, the DT concept includes the comprehension levels of three significant components: a physical object, a digital model, and the communication channel between them [34]. Tao, et al. [35] also introduced data and services as significant components of DT, extending the abstraction level to five dimensions (Figure 3). This 5D construction embodies a dynamic digital counterpart that mirrors physical assets, processes, and systems, potentially facilitating comprehensive monitoring across their lifecycles. DT for SHM purposes involves simultaneous data exchange among the physical and virtual twins and any services carried out. The physical twin represents the tangible, real-world asset with sensors and devices to collect real-time operational and environmental data. The virtual twin is its digital counterpart, created using advanced modelling and simulation techniques to replicate the physical asset’s behaviour, characteristics, and performance. The service encompasses the actionable capabilities enabled by the interaction between the physical and virtual twins, such as real-time monitoring, diagnostics, predictive analysis, and optimisation. This integrated system fosters a continuous feedback loop between the physical and digital realms, facilitating enhanced decision-making, operational efficiency, and effective asset lifecycle management. It is also intended that deterioration and damage to the structure can be detected at their inception through diagnostic procedures such as variation in strain levels or model analysis [36,37].

The operating process of obtaining a physical asset’s condition is measured through IoT and communicated with the digital model to estimate the state of the physical object. From an information modelling perspective, DT is different from the conventional computer-aided design (CAD), which adds more value to model-based systems engineering (MBSE) (modelling to support activities related to system requirements, design, analysis, verification, and validation) by extending the application from early lifecycle phases to operation and maintenance [38].

## 3. Literature Review Methodology

**Figure 4:**
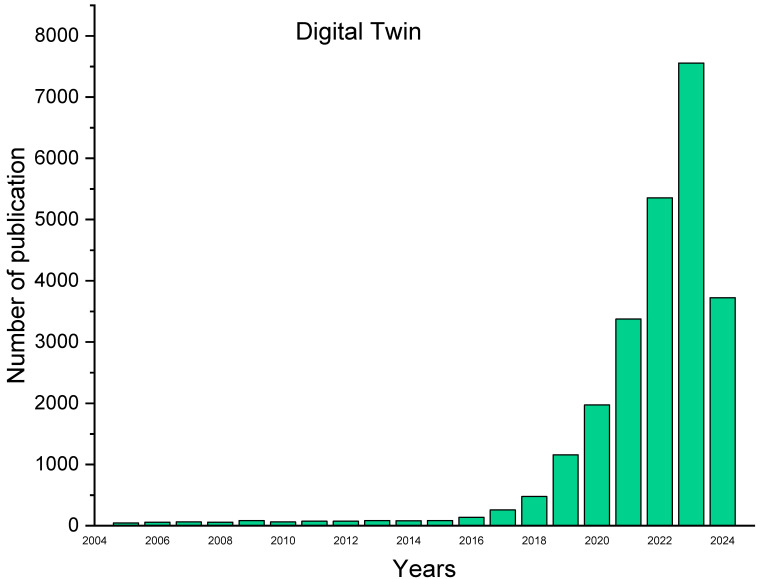
Number of publications versus the years for the search “Digital Twin”.

**Figure 5:**
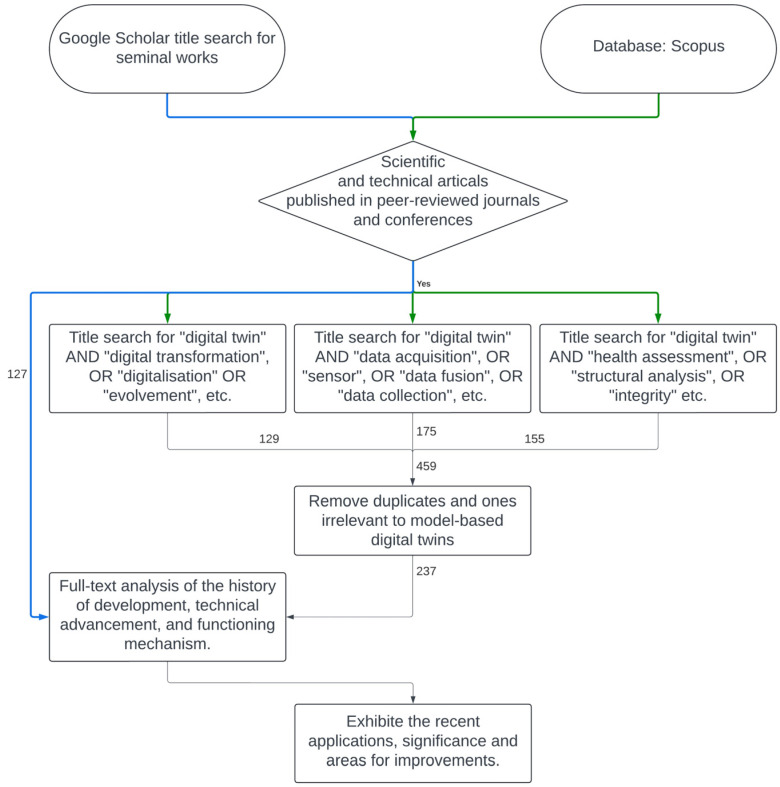
Literature review methodology.

Despite the growing prominence of DT research by using the phrase “Digital Twin”, many published studies were identified (Figure 4). However, limited attention has been given to the most recent applications of DT in the civil infrastructure industry. This review study examines pertinent journal and conference articles published between 2005 and 2024, concentrating on implementation components of DT and their applications for the health assessment of civil infrastructure assets (Figure 5). The reviewing methodology for this study in terms of the search criteria, search strings, and procedure for selecting the available research studies is shown in Figure 5 and Table 2. Three authors independently investigated the databases for the three distinct topics to enhance consistency and reliability, and another author reviewed and commented on the full-text evaluation stage. Then, all the authors contrasted their findings and compiled them. Consequently, more than 400 research studies were initially found. The authors then evaluated the relevance of each research study to the research topic (i.e., applications of DTs in structural health assessment of civil infrastructure). Thus, 184 research studies were incorporated into this review study. The authors read all the included studies to summarise their commonalities and distinctive claims.

**Figure 6:**
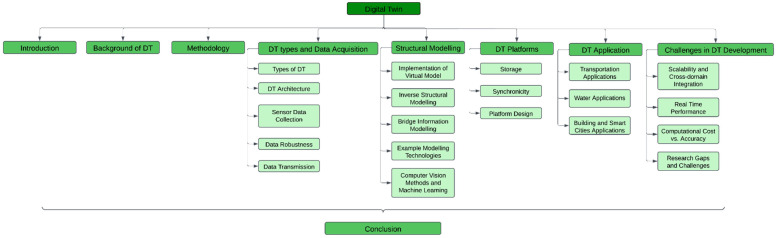
Schematic flow chart of the structure of the study.

Figure 6 outlines the different sections of this review study. Then, we will start with the ‘Introduction’ section, followed by Section 2, which illustrates the methodology of reviewing the literature and filtration for the DT implementation approaches and enabling technologies by previous scholars, including data collection, transmission, and integration methods. The different types of DTs and DT architecture, along with the development footprint of the digital transformation advancement of the civil engineering industry, are discussed in Section 3. The DT applications in civil infrastructures, including the structural health assessment processes and structural modelling techniques, are explained in Section 4, followed by Section 5, which illustrates the requirement of digital platforms and their minimum components for real-time DTs. Section 6 elaborates on potential project risks and DT development and implementation difficulties. The conclusion of this review study explains the importance of DT in structural health management and the future work to improve DT. The findings of this review study are expected to provide insights into DT development and practical implementation in infrastructure engineering.

## 4. Data Acquisition and Transmission

### 4.1. Types of DTs

DTs can be classified into various types depending on the intended functions. Classification criteria include hierarchy, maturity level, and applications. The hierarchy of the DT includes resources combining, sharing, and reusing [39]. DT maturity progresses through five key stages: (1) descriptive, where essential system data are gathered and analysed; (2) diagnostic, identifying inefficiencies and anomalies through model comparisons; (3) predictive, utilising simulations to forecast future behaviour; (4) prescriptive, enabling optimisation and decision-making with actionable insights; and (5) fully integrated, achieving real-time synchronisation and autonomous operations between the physical asset and its digital counterpart. This progression enhances system understanding, decision-making, and operational efficiency [40]. In parallel, the application of the DT varies from the application of the DT system, as it depends highly on the critical requirement for its development to optimise the system’s economic, time, and management [41,42]. The different types of DT are classified as below based on [43,44].

1. DT Prototype: A DT prototype collects the necessary data and information before developing physical infrastructure from the modelled version. This information typically includes CAD drawings, design reports, and, in some cases, the bill of materials. For civil infrastructures, various tests, including destructive tests, are often conducted before constructing the physical twin. Destructive testing is critical in identifying undesirable scenarios and mitigating unpredictable conditions that may be challenging to evaluate using traditional prototypes. Once the DT prototype has been thoroughly validated, the physical twin can be constructed in the real world. This validation process is essential to ensure high levels of simulation accuracy, which directly contributes to the quality and reliability of the resulting physical twin.
2. DT instance: This type of DT is the vice versa of the DT prototype. In these DTs, the physical twin exists, and a shift toward the development of the digitalised twin is needed throughout the life cycle of the physical twin. The process involves transmitting data from real space to virtual space to monitor system performance and evaluate any prediction. It is useful to validate the DT for high-accuracy behaviour and performance of the infrastructure.
3. Performance DT: The process information obtained from minoring the physical twin can be aggregated and analysed to generate actionable data, which can then be used to optimise the structure’s performance in design and maintenance.

### 4.2. DT Architecture

The DT architecture for individual domains varies depending on different factors, including the methods of implementation, protocols to be used, and the persistent DT concept that would be required. This variation is due to the specific information obtained from the DT by the individual domains and the rationality of the DT environment deployment [45]. A general architecture design for a DT mainly consists of three components as described below:

1. The physical world: This is the existing physical twin where the DT will need to be developed. Within the physical world, there are also some essential components that need to be considered, such as the use of the IoT (i.e., sensors), data security, and processing capabilities. In some cases, AI will also be required for big datasets.
2. The virtual world: This is the developed DT itself, which will require major components during development using AI and ML for the DT model. The type of input data obtained from the physical world, and the approach of using the specific type of ML or DML are also essential to be considered as a sub-component within the virtual world component.
3. Connectivity: This is the connectivity between the physical world and the virtual world, which will also need sub-components that require a considerable type of connectivity to be used, such as the use of the Internet, Bluetooth, satellite… etc., or maybe using the cabling network to transfer the data from the physical world to the virtual world (DT).

**Figure 7:**
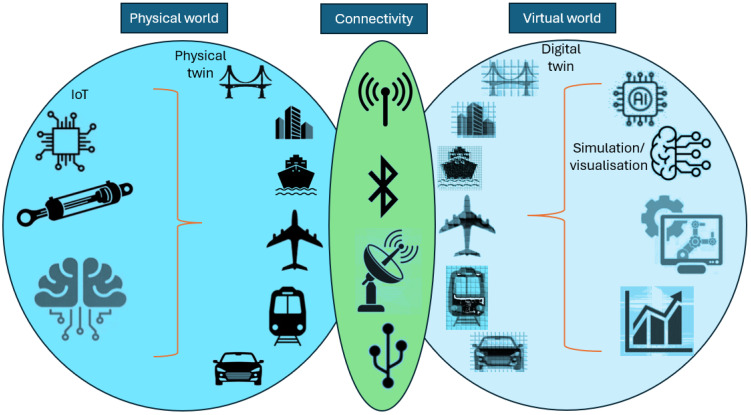
The general architecture of a DT.

An integrated DT architecture may also need to validate the model, power supply to operate the IoT, types of equipment, and data storage capability before and after the virtual world representation. Figure 7 shows typical DT architecture components and sub-components.

**Figure 8:**
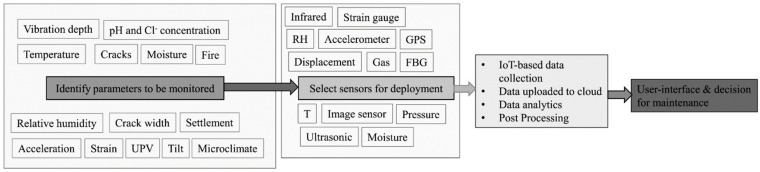
Data collection for DT interpretation.

Sensors have a pivotal role within the context of DTs, facilitating the amalgamation of real-time data derived from physical systems (Figure 8) [46,47,48,49]. They serve as indispensable tools for monitoring diverse parameters encompassing environmental conditions and structural performance. Various strategies are employed to uphold the reliability of sensor data. Integrating data collection, simulation, and interpretation forms the data management chain for any DT workflow. Recent advancements in asset monitoring technologies, coupled with reductions in their costs, have made it possible to deploy sensors capable of measuring a wide range of asset parameters. This capability represents a significant improvement, as such measures were previously either infeasible or unaffordable. The data acquisition phase of the DT process involves selecting appropriate excitation methods, sensor types, the number of sensors needed, sensor placement locations, and the hardware for data acquisition, storage, and transmission. During this process, sensors are typically installed at strategic points on the structure in a non-invasive manner, ensuring no damage to the asset.

### 4.3. Sensor Data Collection

Sensors detect and record various environmental factors, such as temperature, light, motion, pressure, load, and moisture. The collected data can be displayed locally at the sensor or transmitted electronically for further analysis and processing. Key characteristics of sensors include their range, precision, sensitivity, reliability, adaptability to environmental conditions, and energy-harvesting capabilities. Ideally, a sensor should exhibit high sensitivity to the target property while being unaffected by external factors. Sensors are generally classified into two categories: active and passive. As seen in weather satellite sensors, active sensors require an external power source to detect environmental stimuli and generate an output. In contrast, passive sensors operate without an external power source, relying on ambient energy such as light or thermal energy [50]. A glass thermometer, for example, functions as a passive sensor, with mercury expanding or contracting in response to temperature changes.

**Figure 9:**
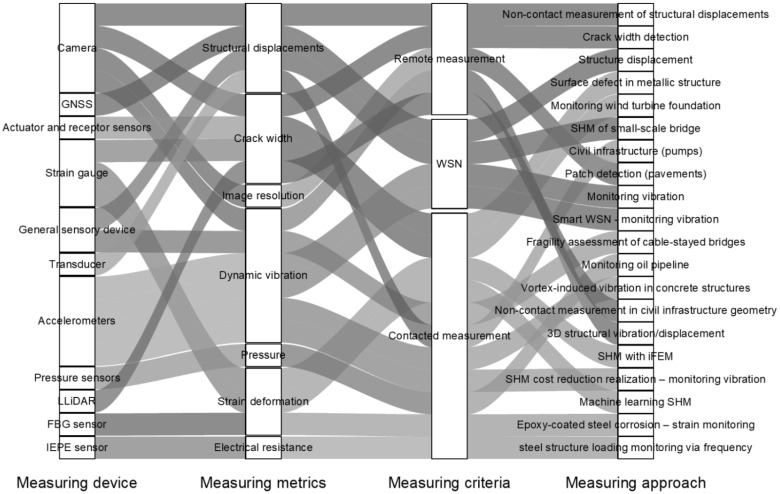
Data acquisition methods attempt (civil infrastructure industry).

**Figure 10:**
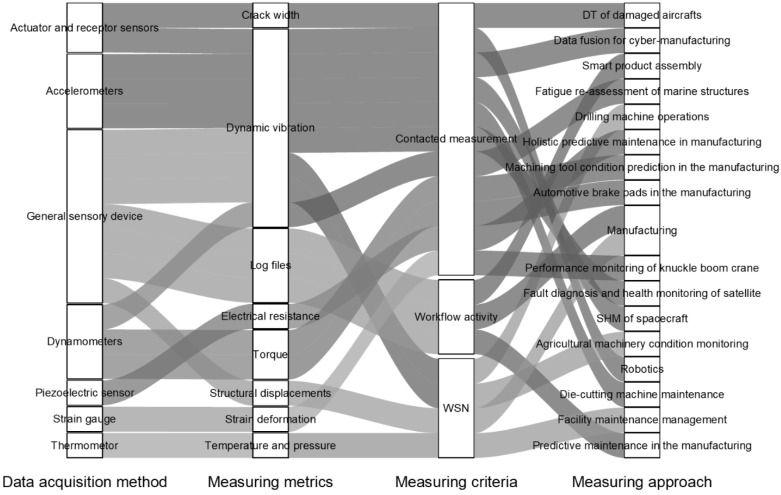
Data acquisition methods attempt (other industries).

A standard DT includes a network of sensors that monitor various parameters within the structure and its environment, such as stress, strain, vibration, tilt, humidity, and temperature. Researchers have widely used advancements in sensor technology to create a range of sensors suitable for continuous data collection. Sensors may be either advanced [51] or conventional [52]. The primary sensors utilised in DT encompass fibre optic sensors (FOSs), accelerometers, vibrating wire transducers, linear variable differential transformers (LVDTs), load cells, strain gauges, inclinometers (slope indicators), tiltmeters, acoustic emission sensors, microelectromechanical systems (MEMSs), and temperature sensors. Figure 9 and Figure 10 provide an overview of the various types of sensors used in recent DT advancements and the particular metrics they can measure by different civil infrastructure researchers [53,54,55,56,57,58,59,60,61,62,63,64,65,66,67,68,69,70,71,72] and other industries [73,74,75,76,77,78,79,80,81,82,83,84,85,86,87], respectively. Middleton, et al. [88] presented a comprehensive review of existing technologies for infrastructure monitoring and stated that, in general, the monitoring data can be classified into four groups: (1) response-based, such as strain, displacement and inclination; (2) geometry-based, such as conventional surveying and laser scanning; (3) vision-based, such as image and video, and (4) loadings, such as operational and environmental loadings.

Whilst DT processes tend to be application-specific, there are inherent drawbacks to the data acquisition units. The deployment of any contact-based sensors is inherently time-consuming. Therefore, the configuration of sensor location and the quantity to be used also need to be optimised to differentiate between areas with higher and lower inspection priorities. The stochastically developed sensor configuration must also consider target structural sensitivity, for sensing meaningful readings for sensor fusion-based structural evaluation [89]. At the same time, there is a lack of reliable performance evaluation metrics among most existing studies related to sensor fusion. More recently, the technological development of WSN and the Industrial Internet of Things (IIoT) have enabled enhancements in integrated sensing and analytics. WSN uses spatially dispersed sensors to monitor, record, and forward structural data to a central location. Thanks to its simplicity of implementation and significant cost reductions, WSN can reduce the installation and maintenance cost of infrastructure [90]. However, data transmission units typically have the highest energy consumption in applications involving wireless sensing nodes. Multiple transmission levels would be required in cases of large-scale infrastructure, adding to the difficulty of accurate synchronisation and a high transmission rate for the data collected to be evaluated simultaneously and centrally [63].

Sensors (stand-alone or combined with other data collection tools) are dominant in collecting data. Other studies on monitoring the remaining useful life of assets include [91,92,93], which used available data from the physical object’s monitoring and control system (inspection records, maintenance work orders, and asset management database) rather than sensors or any other type of extra attachment. Other data collection methods in the literature include 3D digital imaging [71,72,94,95] and ultrasonic imaging [69,96].

### 4.4. Data Robustness

To enhance the reliability and resilience of SHM systems, comprehensive strategies for managing sensor failures and incorporating redundancy are essential. Modern SHM systems rely heavily on sensor networks to provide real-time data on structural conditions. However, the failure of individual sensors due to environmental factors, physical damage, or wear and tear can compromise the system’s overall accuracy and functionality. One practical approach to address this challenge is sensor fusion, which integrates data from multiple sensors of varying types to create a unified and robust dataset. By combining streams of information—such as strain, vibration, and environmental data—sensor fusion mitigates the risks associated with isolated sensor malfunctions and enhances overall measurement accuracy [47,52,97]. For instance, advanced fusion algorithms can integrate inputs from different sensors to provide a more comprehensive evaluation of the structure’s condition, even when some sensors produce incomplete or faulty data [61]. This capability ensures that SHM systems maintain high levels of reliability, even in adverse conditions where localised failures might otherwise compromise the system’s functionality [14].

In addition to sensor fusion, redundancy is a critical factor in ensuring the continuity of SHM operations. Redundancy involves deploying multiple sensors with overlapping coverage areas to maintain system functionality during individual sensor failures. This overlapping deployment creates a backup mechanism, enabling uninterrupted monitoring and data collection [37,67]. When coupled with WSNs, redundancy becomes even more effective, as WSNs dynamically reallocate monitoring responsibilities across the network to compensate for sensor malfunctions [41]. This adaptability ensures that the system can continue functioning seamlessly under challenging conditions, such as extreme weather, high vibrations, or physical disruptions to the sensors [98]. For instance, redundant sensor networks in large infrastructures like bridges ensure continuous data transmission even if specific sensors fail due to localised damage or interference [38]. Furthermore, redundancy strategies enhance fault tolerance, allowing SHM systems to operate reliably in harsh environments where individual sensors are prone to frequent damage while minimising disruptions to system operations [53].

Fault detection mechanisms are pivotal in modern SHM systems to address sensor malfunctions proactively. Powered by AI and ML, these mechanisms analyse historical and real-time data to identify anomalies, such as inconsistent readings, erratic signals, or deviations from expected trends that may indicate sensor degradation or failure [42,53]. Fault detection algorithms use pattern recognition, anomaly detection, and predictive modelling to forecast potential sensor issues, enabling pre-emptive maintenance interventions. This approach ensures that failing sensors can be recalibrated or replaced before they affect the system’s overall performance [99]. For instance, AI-based fault detection systems can monitor vast sensor networks and provide early warnings about sensor health, enabling timely repairs that prevent system downtime [74]. These mechanisms also enhance the reliability of SHM systems by ensuring that data used for structural analysis are accurate and complete, even in partial failures. Predictive maintenance algorithms, integrated with fault detection, reduce operational costs and improve the longevity of the monitoring system by optimising maintenance schedules [58,100].

In addition to redundancy and fault detection, advanced signal processing techniques are indispensable for ensuring data quality and system reliability in SHM applications. Techniques such as Kalman filters and Bayesian networks are crucial in filtering noise, estimating missing data and correcting errors in real time [61,101]. These methods enhance the system’s ability to accurately process large volumes of information, even in environments with high interference levels or incomplete datasets [60]. For instance, Kalman filters dynamically adjust to changing conditions, providing real-time corrections that maintain data integrity over extended monitoring periods [47]. Similarly, Bayesian networks enable probabilistic reasoning to handle uncertainties in sensor data, improving the system’s robustness [14]. When combined with robust hardware designs, environmental shielding, and self-healing technologies, advanced signal processing ensures that SHM systems remain operational and accurate even under demanding conditions [74]. These capabilities are critical in DT applications, where real-time, high-fidelity data are essential for effective decision-making and predictive modelling [37,53].

By integrating sensor fusion, redundancy, fault detection, and signal processing into a cohesive framework, SHM systems can achieve unprecedented reliability levels, adaptability, and resilience. These strategies enable continuous monitoring and accurate real-time analysis and ensure the long-term sustainability of SHM systems, even in complex and dynamic environments. Furthermore, such advancements in SHM technologies directly enhance the effectiveness of Digital Twin implementations, enabling more reliable predictions, better-informed decision-making, and improved maintenance planning for critical infrastructure [41,53,98].

Collecting data from various sensors is the first step in building a comprehensive DT. Once the data are captured, it must be transmitted to the digital model for processing and analysis. This transmission creates a seamless data pipeline, where data flows from the sensors, through transmission systems, and into the DT. The efficiency of this pipeline—encompassing sensor selection, data transmission, and real-time updates—directly impacts the accuracy and responsiveness of the DT. The following section explores the data transmission methods that connect physical sensors to the digital system, ensuring continuous data flow and enabling real-time monitoring and decision-making.

### 4.5. Data Transmission

**Figure 11:**
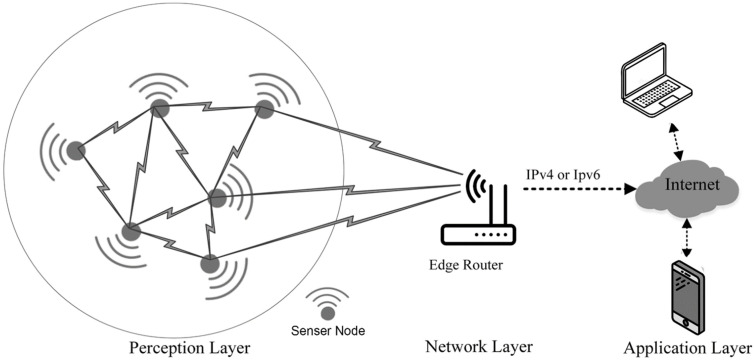
The communication among the WSN layers.

Data transmission systems, include both wired and wireless transmissions. Wire transmission technologies include several approaches, including twisted-pair cable transmission, symmetric cable transmission, coaxial cable transmission, and fibre optic transmission. Figure 11 shows a typical diagram of the wireless transmission system which includes both short-range and long-range technologies. Short-range wireless technologies that are often used include Zig-Bee, Bluetooth, Wi-Fi, Ultra-Wideband (UWB), and Near Field Communication (NFC) [102]. Long-range wireless technologies include several approaches, including GPRS/CDMA, digital radio, spread spectrum microwave, wireless bridge, and satellite communication. Both wired and wireless communications depend on transmission protocols, access strategies, multi-access schemes, channel multiplex modulation, coding and multi-user detection technologies. WSNs play a vital role in the integration of DTs across several sectors. Integrating WSNs makes it feasible to continuously monitor and gather data in real time for virtual duplicates of physical assets or systems. WSNs are used to collect vital information from the physical environment. Afterwards, these data are used to analyse and optimise within the context of the DT. This hyperlink improves the precision and efficiency of DTs by offering continuous updates and valuable insights [100,103,104]. Furthermore, WSNs in DTs enable effective resources allocation, proactive surveillance, and accurate simulation in intelligent grid networks. WSNs enhance operational efficiency and rapid service restoration during unforeseen incidents [105]. Furthermore, WSNs are crucial in assessing the effectiveness of Wireless Software Applications (WSAs) using platforms such as Wireless DT. These systems use DT and real-time ray-tracing technologies to replicate wireless signals and evaluate performance [106].

The sampling rate of the sensor network is another important decision that must be considered. The sampling rate significantly influences the accuracy of performance measurements for physical assets. Tao, et al. [107] successfully elucidated the complexity of selecting a sampling rate in the turbine scenario when they presented their research study. The authors emphasised that monitoring vibration impact from a turbine gearbox at one-minute intervals would lead engineers to detect a superior knowledge of the vibration impact. However, gathering data at a high frequency, such as one sample per second, would also lead to an overwhelming data volume, subsequently causing data transmission congestion. Therefore, the designer needs to carefully select the rate for data sampling from the sensors. Once the designer of the monitoring system has selected the most appropriate sensor sample readings, then the optimal number of sensors to be deployed at strategic locations on the structure would need to be considered [103,108]. Another important decision on the number of sensors is that if sufficient sensors are installed on the structure, they may lead to inaccurate asset descriptions, which are essential for efficient preventive maintenance. Moreover, it might result in inaccurate predictions, impeding any attempts to enhance operational efficiency. Whilst a high quantity of sensors may have a detrimental effect by overwhelming the user with data, a superior knowledge of the structure performance will be obtained. However, this overwhelming data flow may lead to data scattering and place excessive pressure on the software system, potentially causing bottlenecks, delays, and crashes. Therefore, the placement of the sensor network on the Physical Twin is vital as it allows for the retrieval of data that can be compared to the simulated data acquired from the DT [109].

To optimise the effectiveness of a developed DT, it is essential to constantly and accurately update it. The accuracy of an update primarily depends on the uncertainty in measurements, including the performance of sensors, as well as the model and the technique used to infer and update the model parameters based on monitoring data. Recently, several methodologies have been used on a widespread scale, such as the least-squares deterministic model calibration, which is a straightforward and practical approach for concluding. However, this method is prone to overfitting problems in practical engineering applications, where errors associated with measurements and models cannot be disregarded. Therefore, datasets are often small in scale [110]. Bayesian methods may tackle this problem by considering the prior distributions of model parameters. This feature removes abnormal data points in datasets, such as unusual strain readings in distributed sensing [111]. It prevents updating a DT when there are malfunctioning or damaged sensors. It is worth noting that Bayesian methods, such as the Metropolis–Hastings algorithm, often need an iterative process to compute the posterior distribution of model parameters [112]. Over the last decade, machine learning has made substantial progress in its application to data analysis and the estimation of numerical model parameters. Both supervised and unsupervised algorithms have been used. However, the efficacy of unsupervised approaches is now under investigation [97].

Relatively, the update frequency of the DT, sometimes referred to as the refresh rate, is governed by the technology used (such as sensors and computational capabilities of the hardware), the quantity being measured (including the raw volume of sampled data to be processed) and the inference approach applied. Different DT applications need different rates of data updates. To elucidate this matter, Callcut, et al. [113] performed a comparative analysis of the real-time data requirements for several DT applications, such as centralised airport air traffic management, intelligent vehicle navigation systems, and maintenance planning for bridge repair. The first two applications needed real-time refresh rates, whereas the last application did not have a refresh rate. It can be withdrawn that the refresh rate for all DT applications may not be required. Therefore, it can be concluded that the DT should incorporate a new set of distinctive features and crucial values after each significant alteration in the physical asset, in other words, with a frequency that matches the frequency of “significant” modifications on a case-by-case basis [114]. Thus, the ideal refresh rate for a DT does not necessarily occur in real-time but rather at the most appropriate moment to ensure optimal performance. Civil engineering applications requiring tailored refresh rates include structures with complex failure mechanisms, such as shear keys in prestressed concrete components, and buildings in highly seismic regions, where precise monitoring is critical.

Relatively, the refresh rate of the DT highly depends on the type of technology used, the quantity being monitored, and the inference approaches. For the type of technology, the refresh rate of a DT must match the sampling rate of the sensor network. This rate may range from 4000 kHz for accelerometers and 250 Hz for DOFS to one measurement every 15–30 min for robotised topographic stations. It is also crucial to comprehend that the transmission speed of data might fluctuate based on the type of sensors used. The data flow may happen instantaneously with wired sensors, such as distributed sensing. Conversely, wireless sensors, such as accelerometers that use the LoRaWAN protocol, may experience intervals between data transfers that might last for several minutes, and the validity of this material is corroborated by citation [115]. Furthermore, different sensors may acquire data at different intervals, resulting in varied data accessibility. In such a condition, the update procedure may be scheduled to start either when new datasets become available or at certain intervals after all data have been received [116]. Moreover, the analysis may be conducted whenever a new measurement is acquired, contingent upon the model parameter that requires estimation. For instance, compensating for temperature in strain measurements may need to run the analysis constantly. However, estimating vibrational frequency using operational modal analysis may involve waiting for a dataset, which might cause a delay in the refresh rate. The length of the analyses may vary based on the number of iterations required by the inference approach. Thus, a linear model calibrated using the least-squares deterministic calibration method may only need to go through one iteration. A Bayesian parameter estimate that utilises a Markov Chain Monte Carlo simulation typically needs a minimum of 1000 iterations [117].

## 5. Structural Modelling and Health Assessment

DT is a comprehensive system that involves a simultaneous data exchange among the physical twin (geometry, material properties, loading patterns… etc.), its virtual twin, and any services carried out within the two. Furthermore, the collected data from the physical object, as well as simulation data from the virtual model, should be mapped, analysed, and interpreted to inform decision-making on the status of the physical object and optimise its performance. Continuous data exchange ensures the continuous SHM of a structure. It is also assumed that deterioration and damage to the infrastructure can be detected at their inception through diagnostic procedures such as peak strains or model analysis [36,37]. Early deterioration and damage detection will form the basis for intelligent maintenance and rehabilitation-related decision-making. Hence, it is highly demanded that a structural model be incorporated into a DT system of infrastructure to keep track of its structural integrity. Most of the literature refers to only digital models, which visualise only the geometry of structures as the virtual model of a DT system [118]. Several popular commercial software packages such as Bentley 24.00.00.156, ClearEdge3D 5.8.3.0, AVEVA 23.1.000, and Autodesk Collaborate Pro also accommodate only geometric DT. However, a DT can be more beneficial as it is based on a structural model that can provide visualisation of the structural integrity of the existing structure [17].

### 5.1. Implementation of Virtual Model

**Figure 12:**
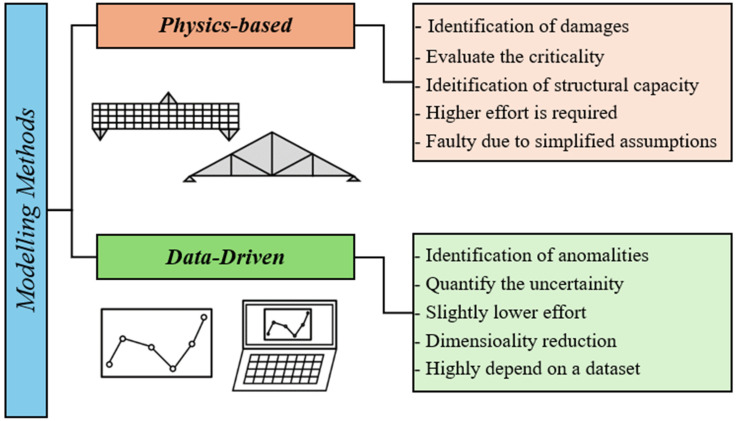
Methods of developing virtual models.

Implementing the virtual model of a DT of a civil infrastructure predominantly depends on its intended usage. Two main approaches to creating the digital replica include a physics-based model-driven method and a data-driven measurement-based method. Figure 12 illustrates a comparison between those two methods. Finite Element Modelling (FEM), Finite Difference Method (FDM) and Computational Fluid Dynamics (CFDs) are commonly adopted methods for physics-based model-driven methods. Physics-based techniques can detect damage and evaluate its criticality, conduct simulations that enable assessing load capacity and the remaining useful life of the asset, and increase the efficiency of health monitoring. The data-driven measurement-based methods include the deployment of machine learning algorithms, time series analysis, data mining, and genetic algorithms; they can also detect anomalies, identify trends and future predictions (in asset behaviour), and quantify uncertainties. Both methods have limitations. For physics-based approaches, challenges include identifying random noise and errors in the acquired data and the inclusion of simplified assumptions in the analysis. Concurrently, the physics-based methods require high computational costs and significant effort and time to be implemented. The Data-driven methods also have limitations, including the difficulties in identifying root causes of identified trends, estimating performance values in locations that lack measured data (e.g., due to lack of access), and the inability to diagnose defects if the data are new and has not previously been included in the training dataset. However, a combination of both methods is found to be superior to utilising those methods individually [17].

In order to determine the appropriate approach that can be used to develop the DT for a civil infrastructure, it is required to consider the expectations of such a model. Ye, et al. [17] discussed several aspects that should be included in a digital replica of a DT of a civil infrastructure. First and foremost, the DT should replicate all the aspects of the infrastructure, including the geometry, material, layout… etc. and a real-time data transfer is required to replicate the continuous occurrences in the existing infrastructure. Moreover, it is required to be equipped with a standard data framework that can be accessed by all the stakeholders and a visualisation platform to communicate the problems to stakeholders in a user-friendly environment.

Also, at immediate sensor network measures of the structural performance that transfer them to the digital model, the DT should be able to virtualise the structural performance simultaneously. It will be challenging to anticipate any structural breakdowns and trigger warnings for infrastructure controllers by comparing calculated responses to measured readings at the same time; therefore, a continuous recording of the DT must also be obtained for any evaluation of the historical performance [17,119]. Another concerning factor for DT of the infrastructure is that the DT should be trained under real-world circumstances prior to the application in the real world. An applicable approach to train the DT models would be to measure the structural performance of the infrastructure over a set time and send these data to the DT. However, flaws in measurement instruments, environmental factors and storage device errors could also cause the measured data to be dispersed [120]. For instance, the influence of ground vibrations at the interface between a structure and soil depends severely on the soil and the structure interaction. As a result, Foundation Input Motions (FIMs) must be included in the DT model [120], and DT must be responsive to actual environmental factors. Hence, technology should be used to merge the real environment with the created models [121].

### 5.2. Inverse Structural Modelling

The preparation of the virtual models should analyse the data that is acquired by the data acquisition systems and determine the overall structural integrity of the interested structure. In addition, the general forward process of the overall structural response considering the loadings and boundary conditions that are acting on structures must be determined [122]. Hence, the loading-to-structural response process is inversely proceeded and, therefore, inverse structural modelling is expected to be performed by the virtual model. However, inverse methods are highly sensitive, where small variations in the acquired data can cause significant deviations in structural response predictions. For instance, for bridges, the researchers can consider the vehicular loads as the only load that is applied to a bridge and measure that to develop the digital model and to make a forward problem out of this [17,119]. However, some of the inverse methods are also in practice. For instance, the inverse load identification method developed by Gupta [122] considers the linear proportionality between the load and strain, and the linearly behaving nature is expected at the interesting location of the structure. This method can work with minimum obstacles only with plate/shell elements. Wolf Star Technologies has also used a similar method and implemented a software package for this purpose, which consisted of three software programmes, namely “True Load”, “True QSE” and “True LDE”. The True load software can turn different parts of a structure into load transducers, and it suggests the most suitable places to install strain gauges. Then, the existing structure is also instrumented with sensors at the same location, when the measurements of those strain gauges are fed into the true load software. It calculates the applied load on that structure. Subsequently, the load calculations of True Load can be transferred into True QSE, and structural behaviour will be produced. Also, True LDE can be used for Linear Dynamic events. However, this software does not support the beam, column, or truss elements.

Since most of the inverse methods are highly ill-conditioned, even due to unavoidable or minor errors of the measuring devices, a demand arises to investigate a more stable and accurate method to sense and determine structural health. Shape sensing has been introduced as a novel technique for this purpose, and it can be explained as the real-time process of estimating the deformed shape of a structure based on measured strain data, which effectively responds to both static and dynamic cases [123]. Strain responses reflect structural behaviours more comprehensively and accurately as they are influenced by higher modes at low frequencies. Hence, shape-sensing techniques can accurately simulate the effect of higher modes when compared to the acceleration responses [122]. Solutions for shape sensing have been proposed under two algorithm types: modal/analytical/curve fitting approaches and the inverse finite element method (iFEM) [99]. Table 3 shows the methods used for the shape estimation of structures.

**Figure 13:**
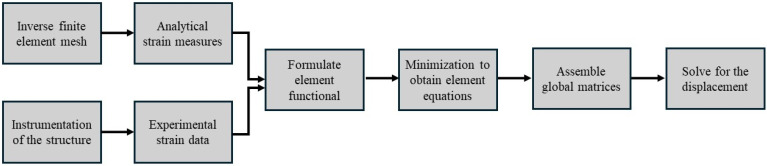
Systematic workflow of 2D iFEM procedure.

The inverse finite element was identified as the most suitable method for shape sensing in most of the research. Hence, it was reviewed in more detail for the current study. The iFEM was first proposed by Tessler and Spangler [133]. iFEM is a high-potential function that is capable of monitoring displacement and the stress/strain of engineering structures, and it is based on the minimisation of the weighted least square functional between the experimentally measured and analytical strain values of a structure [134]. It can be applied to estimate the shape of complex structures under both static and dynamic loads. Also, material properties, loading details, and damping characteristics of the structure are not required for iFEM. In the direct FEM, elements shape functions will be used first to obtain the analytical strain values, then, the strain-displacement relationships will be used for implementing the formulation. Ultimately, the displacement field of the structure will be achieved by solving a set of linear algebraic equations. However, this formulation has been limited only to 1D and 2D cases, such as beams/frames and plates/shells, respectively. Figure 13 illustrates a systematic workflow chart of the 2D iFEM.

Gherlone, et al. [135] applied Timoshenko beam theory to propose a C0—continuous inverse beam element, which was tested with simulated strain data [136] and validated on a cantilever beam under static and dynamic loads. Their study also accounted for torsional, bending, and transverse shear strains in simple beam cross-sections. However, this method required two or more nodes to accurately predict the transverse displacement field, and the effect of the missing strain data has not been addressed. Investigations were further conducted for inverse beam elements, and this approach was extended gradually for different scenarios. For instance, Zhao, et al. [137] attempted on beams with variable cross-sections, whilst Chen, et al. [138] attempted on complex loading conditions. The inverse beam element was not only based on the Timoshenko beam theory, but later investigations also introduced it using the Euler–Bernoulli beam theory as well [139]. You and Ren [140] further developed this method in order to avoid singular matrices.

In addition, the iFEM formulation mainly depends on the shape functions of a particular element type, as the first step of the investigations in the iFEM series, Tessler and Spangler [133] introduced a 2D three-node inverse element (iMIN3), which has been validated experimentally. The iMIN3 was based on the Mindlin theory [141], and continuous shape functions were used for the formulation [142]. Also, the four-node inverse quadrilateral element (iQS4) was introduced in [143] with its validation, and Kefal [144] introduced an eight -nodes element (iCS8), which acts as a curved shell element for better simulations of iFEM. The 2D iFEM has been widely used by researchers for diverse structures such as wing-shaped plates, panels, wing boxes, carriers etc. [145,146,147,148,149,150,151].

The iFEM method has been used for SHM by different researchers, for instance, Vazquez, et al. [152] and Quach, et al. [153] used Fibre optic sensors and iFEM methodology for the crack detection of a laboratory-scaled beam model. Colombo, et al. [154] attempted to conduct an anomaly detection of a structure by using an anomaly detection index. The authors compared the reconstructed strain, which was obtained from iFEM, together with the measured strain by sensors to determine the condition of the structure. Li, et al. [155] investigated the possibility of using iFEM for the damage identification of wind turbine structures using four different damage patterns, and concluded that iFEM is able to identify the damage notwithstanding the location and the size of the damage. Also, Li, et al. [156] extended iFEM for the damage identification of offshore wind turbine structures as well. Likewise, iFEM has been used for many applications in the industry.

While iFEM is a favourable method due to its functionalities, there are several research issues related to its implementation. One of the major drawbacks is the requirement for a large number of strain gauges. Generally, iFEM needs many FBG strain gauges for accurate estimations. However, FBGs are expensive and require a cautious installation procedure in existing infrastructure due to their brittleness sensitivity [157]. In addition, the FBG sensors are required to be installed on the top and bottom surfaces of the structure to carry out the iFEM analysis, as the structure is subjected to different loading patterns throughout its lifetime [158]. This method is unsuitable because FBG sensors are highly vulnerable to damage from continuous vehicle movements. Additionally, iFEM has been primarily limited to 1D or 2D simulations. While it is capable of real-time analysis, it does not fully meet the requirements for DT implementation. The literature reveals only a few studies that have applied iFEM to full-field displacement analysis of large, complex structures with diverse sections and composite materials. Real-world applications of this method for real-time SHM are rare, raising concerns about its effectiveness for SHM of concrete infrastructure.

### 5.3. Bridge Information Modelling (BrIM)

Building Information Modelling (BIM) was the first modelling workflow used for detailed interdisciplinary 3D building models. The workflow is dedicated to the creation, sharing, exchanging and management of building-related information. Simply, the building data are stored and managed in BIM, and this level of comprehension has now transferred across bridges, as well where that system is known as BrIM [159]. The main purpose of a BrIM is to implement a DT of a bridge by feeding real-time data into BrIMs. Adibfar and Costin [160] attempted to implement a DT model for a prototype bridge by inserting Weigh-in-motion (WIM) data into a bridge model. Arduino sensor systems were used to capture the weights of vehicle models. The technology of this system recognised overloaded cars and warned the controller before the vehicle approached the tested bridge. Dan, et al. [119] attempted to implement DT systems for bridges with measured traffic loads that are situated in regional areas. WIM or multi-source heterogeneous machine vision techniques were used for the purpose of measuring the traffic loads. This system was separated into three segments with the aims of monitoring traffic load, monitoring real-time conditions and warning about extreme loads in working conditions. The authors concluded that DT models, modelling techniques and load-measuring techniques have been validated for a group of real bridges in Shanghai, China. It was demonstrated that employing measured traffic loads to obtain the interaction between the physical bridge and the digital model is the most straightforward and sensible method.

Kaewunruen, et al. [98] integrated BIM with bridge risk inspection model in a unified information platform (BIM+) to facilitate risk-based maintenance planning in prone to extreme conditions environments. Their proposed model combined technologies such as models and spatial positioning to offer a comprehensive information solution for risk data collection (monitoring module), analysis (data analysis module), and sharing (data sharing module). Their proposed method could obtain the results of analysis (by the method) which were then sent to related individuals in the form of charts, data… etc. to decide about the risk treatment. The authors used Revit 2018 to build a 3D model and scanners to scan cracks analyse them through concrete multi-function tester (SCC-MATS) produced by Sichuan Shengtuo Testing Technology Co., Ltd. (in Chengdu, China), which can be integrated with BIM to obtain crack information. The authors then upload the results into the BIM model to visually present the crack location, compare its extent to the acceptable value and list it for future maintenance. They also used CSI-Bridge 2015 to simulate bridge deformation, force, and stress because of natural hazard risks (caused by environment, geology, hydrology, and meteorological conditions). The CSI-Bridge was also used to simulate overload and determine the destructive passing vehicles on the causing risks. Their study proposed to adopt a multi-neural network model and a multi-element mapping mathematical model to conduct early safety warnings.

### 5.4. Modelling Technologies for DT of Bridges

Table 4 shows the modelling techniques and tools that have been used by different researchers for structural analysis and monitoring of in-service bridges in the civil engineering field.

From Table 4, it can be noted that the FE modelling was identified to be the most popular model development technique; however, real-time FE models of the interested bridge structure were not developed by any of the studies. This could be due to the high computational cost; it is extensively challenging, and even challenging for a supercomputer. However, in that case, it is recommended to lower the complexity of the FE modelling by following several techniques such as Mass-Spring Systems [165], Model Order Reduction Techniques [166], Adaptive mesh refinement [167] and FEM-based training of Neural-Networks [168]. The applications of such methods are seldom seen in bridge DT systems. Also, most researchers have used previously collected sensor readings on FE models to monitor structural behaviour, whilst these monitoring processes have been limited to a small section of a bridge (beam, girder, slab… etc.). A real-time FE model for the structural integrity assessment of the whole bridge was not developed. Also, several studies have focused only on displaying sensor readings in real time and obtaining maintenance decisions by introducing a threshold value. Also, the smooth and real-time connectivity between the virtual model and the actual structure should be considered thoroughly when developing DT systems. Some data acquisition units are equipped with the facility to obtain the measurements from the structure and upload it into their cloud service at the same time. In that case, the connection can be easily implemented using a programming language like MATLAB or Python. Apart from that, cyber-physical synchronisation technology can also be identified as a potential method to facilitate the connection between the physical infrastructure assets and their replicas [79]. However, this system has not been used for bridge infrastructure.

### 5.5. Computer Vision Methods and Machine Learning

In addition to physics-based modelling techniques, computer vision (CV)-based methods represent a significant mainstream approach in SHM. Since the 1990s, CV has been widely employed for measuring object displacements [169]. A key advantage of CV-based methods lies in the ease and safety of acquiring images and videos, particularly in scenarios that demand rapid response, such as emergencies. Unlike traditional sensor-based approaches, CV systems require minimal installation or setup, making them highly practical for such applications. Furthermore, CV techniques excel in full-field measurements due to their high-resolution capabilities, as each image comprises millions of pixels embedded with rich information. This enables full-field displacement measurement, where the movement of all parts of a structure can be captured, offering a comprehensive understanding of its health condition. In practice, object displacement in CV is typically determined through the detection and tracking of key points, with standard approaches, including full-field 3D vibration displacement measurements [170,171,172,173,174].

Despite these advancements, the integration of machine learning (ML) in SHM studies remains limited, highlighting a notable research gap. Early CV methods, prior to the widespread adoption of ML and Deep Learning (DL) technologies in the 2010s, primarily relied on deterministic algorithms and explicit mathematical models for image processing and pattern recognition. These approaches, while effective in controlled environments, struggled with the complexities of dynamic, real-world scenarios, such as construction sites [175].

Since the 2010s, CV for SHM has seen significant progress due to the incorporation of ML, particularly DL, enabling it to tackle complex challenges beyond laboratory settings and expanding its application across diverse engineering fields. Reviews on the application of ML and DL in SHM, include [176,177,178,179,180,181,182]. ML and DL have revolutionised the ability to model dynamic environments characterised by numerous variables. However, despite these advancements, there is still a research gap in fully leveraging ML techniques in current SHM studies. Many existing approaches continue to rely on traditional CV methods or use ML in a limited capacity, particularly in areas such as data integration, scalability, and real-time analysis. Addressing these gaps presents opportunities for further innovation in SHM, especially in developing robust, adaptive, and fully automated monitoring systems.

## 6. DT Platforms

DT Platform is one of the major components in the development of DT. The computation of the DT can require extensive decision-making on the selection of the type of AI and typical skills together with IoTs or image processing; however, there is a need for platforms that act as a connector between the physical and digital worlds. Therefore, a complete DT can be obtained. A complete DT platform has the potential for data management by storing and synchronising real-time data. The intelligent platform can also be used to translate the data to an interpretable format for the application of DT processing. Another benefit of the platform is that it can act as a mother to subsequently feed the data to the DT by queueing the data, typically when a large number of data are collected in very short time intervals. For this purpose, the DT is structured and accessible when it is required.

### 6.1. Storage

Data storage is one of the most important components within the DT platform as the real-time data have a specific lifetime. Thus, the real-time data obtained from the physical world are fed into the digital world and dismissed; it is then impossible to evaluate any historical importance of the data. Storing the data within the platform while the data are continuously fed into the digital world will lead digital owners/asset owners to revise the history of the structural performance at a desired time. Another benefit of data storage is the learning approach toward the design of new constructions [183]. The designer can refer to the historical data of the structural performance during the designing process for the improvement of the structural performance/strength. The storage requirements for DT platforms depend on the type and frequency of data collection, often necessitating large-scale cloud solutions. Relatively, secure data storage management is required with the permission of specific avatars to have access to these data with specific details of whom, when, and the history of the browsing toward the data storage [45]. It is also very important to have a backup of the data storage to ensure that these data are kept securely in case of any technical issues that may occur [184].

### 6.2. Synchronicity

Synchronisation ensures that the DT reflects real-time performance, enabling event analysis, predictions, and seamless data integration. The synchronisation of the platform is another crucial component within a complete DT [185]. The frequency of synchronisation implies the change in the DT world. A high frequency of synchronisation represents a superior real-time DT world. The most beneficial aspect of high-frequency synchronisation includes higher management of the data within the platform when storing the measurement data (i.e., from IoTs) and the notification for the asset owners/avatars for real-time DT [186]. Also, at a higher rate of synchronisation, the smaller data pack of storage can be stored within the platform, leading to a much more efficient platform functionality. For instance, the data collection from ten sensors is much greater in comparison to the data collected from two sensors only, and by considering the same rate synchronising the platform, then there is a possibility that the functionality of the platform could be lower for the ten sensors’ data collection in comparison to the two sensors scenario. Platform functionality may fail when large volumes of data are collected at a low synchronisation rate, during the loading of the queueing of the data feeding to the platform. Subsequently, with the higher rate of the synchronisation of the platform; therefore, by feeding the real-time data to the digital world model, the asset owner/avatar can determine the critical performance of the physical world with minimum time delay. Therefore, processing efficient synchronisation obeys the design principles of the platform in terms of reliability, scalability, and data orientation for correct functionality of the platform [185].

### 6.3. Platform Design

**Figure 14:**
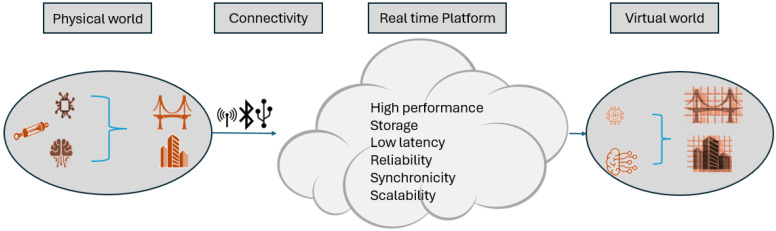
Typical platform design components.

The development of real-time intelligent platforms is necessary to refine key processing such as capturing data, data persistence, and orientation. The ability to capture the real-time data via the different connectivity methods must be configured from the developed platform whether this would be through wireless or wired techniques. It is crucial that the platform is compatible with the format of the data collected (i.e., IoTs). Whereas this is not achievable, the platform must be designed to interpret data to the required format that would be fed to the digital world model. Also, the platform is required to recover properly where at any time a failure occurs within the platform, and they will require high performance to recover time loss and data feeding to the digital world for a real-time operation (i.e., indicating there is no failure occurred). Alternatively, the storage as discussed in earlier sections is the crucial component of the platform whereas substantial storage is required for the data collection, and system operation (i.e., for ML and AI) and offers a low latency minimising the trafficking during the operational process at a high data volume transitioning. In addition, the reliability of the operation and capability of delivering the data within feeding to the digital world are also important components of platform design. The synchronicity of the platform for a real digital world is also crucial (i.e., discussed earlier) while the scalability to convert, interpret, and integrate the data to close the loop is another major component of the platform to communicate with IoTs and the scripting of the AI. The development of the platform is highly dependent on the application together with the amount of data to be collected at different frequencies and/or real tile applications, therefore, the designer can consider a simple or a much-complicated platform for typical applications. Figure 14 shows the typical design components of the platform.

## 7. DT Application

**Figure 15:**
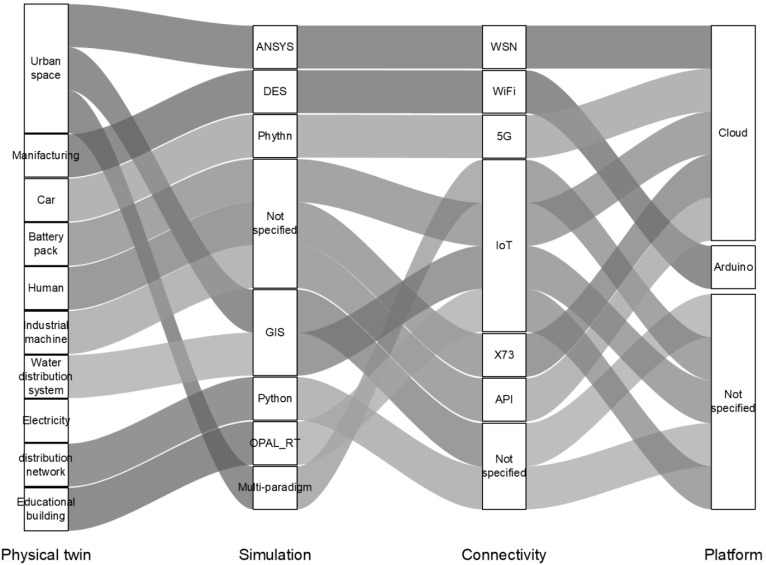
DT development for various types of applications.

DT development has been used for various types of applications such as the automotive, urban space, industrial machines, etc. Figure 15 shows the implementation of the DT in different fields and the types of applications of the platforms that have been used accordingly [186,187,188,189,190,191,192,193,194]. However, the implementation of the DT in the civil engineering field is yet to be limited, and it is in the initial stages. Generally, in the civil engineering field, the DT is developed for monitoring structural behaviour and asset conditions as a replacement for the traditional visual or NDT inspection, as mentioned earlier. Mahmoodian, et al. [11] developed a DT for a section (i.e., steel truss) of port structure (Dalrymple Bay Coal Terminal in Australia) as a case study to monitor the structural behaviour and evaluating the multi-criteria decision-making by using the Fuzzy method. The authors used the ANSYS 2024R2 software to build the digital world of the structure together with IoTs for data reading of the physical world. To link the two worlds together (i.e., physical and digital), the authors used ThingsWorx as the platform. The platform operated in real-time data formatting and scheduling while the digital world of the support structure was visualised. Pregnolato, et al. [195] also developed a DT for the tower saddles of the Clifton Suspension Bridge in the United Kingdom to monitor the performance of the tower saddles. The authors used Midas V945 software to develop a digital world structure that visualised and quantified the structural performance based on the readings measured from the IoTs. The authors used the MQTT message broker as a link and platform to monitor and mirror the physical and digital worlds. While there is limited research work on the platform development for the DTl; however, the concept of the DT is currently an attractive topic and researchers are working in great effort in the development of the DT by using different approaches such as 3D laser scanning, images, and point of clouds where the platform are generally hardware systems, which limit DT operations on a real-time basis [16,118,196].

### 7.1. DT Applications in Transportation Systems

Over 1.24 million deaths and 50 million minor injuries are being reported worldwide due to road traffic [113], of which 1123 cases (both deaths and injuries) are inside Australia [197]. Hence, it is crucial to secure the safety of the overall transportation system of a country. Several shortcomings, such as complex traffic congestion, slow performance, a high level of carbon emissions, and inefficient maintenance practises, are present in current transportation systems due to increased urbanisation, emphasising the need for an upgraded and digitalised transportation system [113]. Consequently, they paved the way for the utilisation of Intelligent Transportation Systems (ITS) incorporating the DT concept together with ML and DL technologies [75]. However, the DT concept has not been extensively implemented in ITS, and the majority of the possibilities are yet to be discovered [198]. The DT concept has been utilised in all three modes of transportation, including land, air, and water, to optimise the facilities [199]. A DT is capable of enhancing the current transportation systems by improving road safety, aiding in mode selections, providing a digital identity, simulating traffic in real time, and optimising the cost of operation and maintenance [198,199,200]. Additionally, the usage of ML and DL will assist in all operation-related decision-making strategies, such as reducing the waiting time of vehicles at the traffic lights, effectively managing the traffic flow, forecasting any possible traffic congestion, providing safe driving guidelines, etc. [198].

One of the most standard platforms that utilises the characteristics of the DT concept is Google Maps [113]. It provides users with the shortest route to their destination by analysing real-time traffic data collected from smartphones. Rudskoy, et al. [201] discussed an ITS model that has been developed to enhance planning and decision-making on key roadways in Western Australia. This system consists of a DT of transport infrastructure and is capable of displaying real-time traffic conditions with sensors that are positioned on roads. Additionally, it enables traffic forecasting, traffic simulations, and planning for the expansion of the road network with AI algorithms. Similarly, the ‘SAVe’ project focused on implementing a DT for urban transportation systems in Ingolstadt, Germany. Guo, et al. [202] suggested a 3D DT model for Corporate–Vehicle Infrastructure System (CVIS) where LiDAR (Light Detection and Ranging) devices were used as the sensing device and 3D model visualisation was performed in LGSVL (a high fidelity simulator for autonomous driving). This system was able to capture the dynamic response of the objects. However, the quick changes in the objects could not be identified accurately with this system. Wang, et al. [203] investigated the possibility of model construction for highway traffic utilising 3D geographic information system (GIS) technology to implement a DT. CAD drawings that include road designs and route planning were used e to digitise using GIS effectively. However, they have highlighted the importance of enhancing the verification of the DT system, life cycle, and framework of highway traffic while suggesting a feasibility analysis regarding this.

Adibfar and Costin [160] attempted to implement a DT model for a prototype bridge by inserting weigh-in-motion (WIM) data into the bridge model. Arduino sensor systems were used to capture the weights of vehicle models. The technology of this system recognised overloaded cars and warned the controller prior to the vehicle’s approach to the tested bridge. Dan, et al. [119] attempted to implement DT systems for bridges with measured traffic loads that are situated in regional areas. WIM or multi-source heterogeneous machine vision techniques were used to measure traffic loads. This system was separated into three segments with the aims of monitoring traffic load, monitoring real-time conditions, and issuing warnings about extreme loads in working conditions. Further, DT models, modelling techniques and load-measuring techniques, have been validated for a group of real bridges in Shanghai, China. It was demonstrated that employing measured traffic loads to obtain the interaction between the physical bridge and the digital model is the most straightforward and sensible method [119].

In a unified information platform (BIM+), Kaewunruen, et al. [98] integrated BIM with a bridge risk inspection model to facilitate risk-based maintenance planning in environments prone to extreme conditions. Their proposed model combined technologies such as models and spatial positioning to offer a comprehensive information solution for risk data collection, analysis, and sharing. Their proposed method would have the results of the analysis sent to related individuals in the form of charts, data, etc., to decide about the risk treatment. They then upload the results into the BIM model to visually present the location of the crack, compare its extent to the acceptable value, and create a maintenance list. Another risk category in their study was called human risks and included collision, overloading, explosion, etc., which were measured by sensors (in the case of load, for example). CSI-Bridge was used to simulate overload and, if destructive, to warn passing vehicles about the bridge’s risk. Their study proposed to adopt a multi-neural network model and a multi-element mapping mathematical model to conduct early safety warnings.

The application of DT is observed in maritime transportation as well. For example, Liu, et al. [204] investigated the security of DT-equipped maritime transport systems, and the DT implementation was based on relay cooperation IoT. It was discovered that the DTs that were implemented could improve communication by extracting energy from interference data. The likelihood of an outage and the efficiency of data transmission using DTs were further examined. As a result, it was discovered that a highly successful transmission probability reduces transmission delay, whereas outage probabilities are dependent on relay numbers. The importance of DTs is further emphasised by examples from air transportation. A conceivable use of DTs is air traffic control, which will enable effective traffic flow control by recording real-time data on aeroplanes [113]. The US Air Force also cites DTs as part of their long-term strategy, with the eventual goal of implementing a DT for each aeroplane for SHM purposes [38].

### 7.2. DT Applications in Water Systems

A water distribution network is responsible for supplying water of acceptable quality and quantity for daily consumption, and hence, it plays a vital role in maintaining the continuity of daily activities. Irrigation assets are another important area for the region’s economy. Relevant asset owners like Goulburn Murray Water, which manages around 50% of Victoria’s groundwater, need more intelligent systems to provide sustainable service to their customers [205]. Rising population and water demand necessitate frequent monitoring of distribution networks, timely repairs, and efficient leak detection. These systems are complex in nature, and it is not possible to manage them manually [206]. For instance, urban water treatment plants comprise a huge number of processes and are the roots of an urban water distribution system. Nowadays, automated systems are being used for handling those systems. Further, smart water grids (SWGs), a system that includes smart pipes and sensors, smart water metering, a GIS, and supervisory, control, and data acquisition (SCADA), is also a novel concept in water management that is currently being used [207].

The involvement of DT may enhance the productivity of water distribution and water management systems as it allows supervisory control, damage detection, monitoring of internal pressure, predictive maintenance, etc. It is common to see DTs at the single component level of an urban water distribution network. For instance, pump manufacturers implement DTs of pumps to monitor the operating parameters and enhance pump productivity [208]. Further, a DT should mimic the path of a water drop for its entire path from a water body to a water tap, and a demand for a DT exists at the system level to virtually simulate the whole urban water system. However, it is rare to see such studies on DTs, and most of the researchers have focused their attention only on DTs for water distribution networks [191]. Various attempts by researchers to incorporate cyber-physical systems (CPS) and software (EPANET), water distribution system modelling software) paved the way for the implementation of DT for water distribution systems (WDS). Those systems allowed to optimise the water distribution based on the availability and demand [209], thereby monitoring the behaviour of water quality in WDS [210,211] and fault diagnosis based on the real-time data [212].

The first DT for a water utility was developed for the WDS in Valencia, Spain, where 1.6 million people used water from this system [191]. This DT system was able to optimise the network design based on future demands to plan upcoming extensions or replacements, detect faults in the system, analyse the operating parameters on a daily basis, indicate warnings and emergencies, and forecast the water quality parameters. Yet, it was difficult to synchronise this model with real-time data due to its size, and a more simplified version of this setup is suggested. The model that accounts for the water quality of the system has not yet been implemented. Similarly, a DT was used for a district-metered area in Lisbon, Portugal, to facilitate the drinking water system of that area [207]. It was found that the DT system can identify the leakages and the need for repair immediately, resulting in water savings of up to 15%. Ramos, et al. [207] also highlighted that DT could positively impact the water economy by ensuring lower energy consumption, high environmental protection, and satisfied consumers. Further, DTs are being practised by several utility companies all over the world, such as Consorci d’ Aigües de Tarragona (Spain), Halifax (Canada), Portsmouth Water and Anglian Water (UK) for leak detection and fault diagnosis [206].

Wang, et al. [213] also attempted to use the concept of DT for hydraulic system fault diagnosis. They implemented the DT model in three major steps: initial model fitting, fault diagnosis, and predictive maintenance. The virtual model of the actual system was implemented, tuned with the actual model for less than 15% prediction offset, fed the common fault modules to the system, and then used for predictive maintenance to monitor the variation in certain parameters. It was found that this DT model can predict the faults with up to 89% accuracy, which is 9% higher than a contemporary simulation model. According to Pedersen, et al. [206], VCS Denmark, a utility company, attempted to use DT for an urban drainage system beginning in 2008 to identify the behaviour of a drainage system by monitoring the extracted data from several rain gauges. Both the sewer and stormwater systems were included in that system. However, the model could be run only once per day, and it took around two hours to run the model. It has been reported that this system was updated to simulate the infiltration inflow and automatically update the virtual models.

### 7.3. DT Applications in Buildings and Smart Cities

The purpose of using DT for buildings is to implement a 3D digital model to a well-defined connection with its actual building that is sensitive to time. Consequently, a Building Digital Twin (BDT) is much more capable of enhancing the functionality of different phases of Building Lifecycle Management (BLM), which includes the planning, designing, constructing, operating, maintaining, and demolishing of the building [214]. Khajavi, et al. [215] have explained that data components from BIM, wireless sensor networks (WSNs) that produce real-time data, and data integration/analysis through ML algorithms make a complete DT of a building.

Table 5 outlines recent BDT applications in several phases of BLM.

Apart from the life cycle phases of a building, [225] implemented a BDT combining the concepts of CV and BIM. This system was a combination of object detection from surveillance video, a camera-BIM location transformation, and object matching. It first identified the object by analysing the video stream, and subsequently, a 3D object detection network estimated the locations and rotations of 2D images. Following that, the camera video was transformed into BIM, and finally, the objects in the BIM were updated regarding the transformation. The accuracy of this system has also been validated with prediction offsets of less than 0.2 m, and authors have highlighted the importance of this system for the operational and maintenance activities of buildings. Nevertheless, this was capable of only visualising the real building in a virtual context.

The concept of “smart cities” catches the attention of the modern world due to the advancements in modern technologies. Smart cities use computational facilities and improve the interaction between each element to optimise the city’s available services. The DT concept also works with smart cities and enables augmenting community services. For instance, Virtual Singapore, well-known as the “Digital Twin City”, created by Singapore public authorities, is an interactive 3D city model that virtually simulates each part of the city [226]. They have even investigated preparing biologically sensitive 3D models for trees to be presented in the virtual city [227]. Hence, city planning will be at our fingertips soon, and DT cities will be able to serve the entire community effectively. Further, the Data for the Public Good report published by the National Infrastructure Commission in the UK has highlighted the significance of data sharing to enhance the performance of infrastructure while suggesting the need for a “National Digital Twin” for the betterment of infrastructure performance. Later, the Digital Framework Task Group (DFTG) in the UK also published “Gemini Principles” to guide it [228]. Lu, et al. [229] also prepared a DT-related anomaly detection system for the West Cambridge site of the University of Cambridge in the UK.

## 8. Challenges in DT Development

The application of the DT is monitoring and collecting real-time data which are fed into a digital system for analysing and evaluation purposes (i.e., assessment of current conditions to predict its future trend) [101]. To develop an indistinguishable DT based on physical structure, there are several challenges that designers of a DT may confront in different areas. One of the major challenges in the development of DT is the transformation of the measurement data into the virtual world by using IoT devices (i.e., sensors, actuators, and appliances), which involve several steps of communication processing, the development of simulated DT, its validation, and real-time DT (i.e., perfect DT). To develop the real-time DT, some of the major challenges that current DT research may confront are further explained in the following.

### 8.1. Scalability and Cross-Domain Integration

Digital Twin (DT) systems face significant scalability challenges, especially with the exponential growth of data from Structural Health Monitoring (SHM) systems due to advancements in sensor technologies and IoT integration. Managing these data efficiently requires robust storage, retrieval, and processing mechanisms, yet current systems lack scalable data pipelines for real-time synchronisation. This limitation reduces the applicability of DT systems to large-scale infrastructure projects, necessitating innovative strategies for data management [6,8]. The challenge of identifying structural members becomes particularly relevant for large-scale infrastructures, where selecting the right DT elements must balance cost and structural requirements. Older infrastructure further exacerbates these issues, as the absence of historical digital data often necessitates extensive and resource-intensive data collection efforts to replicate deteriorated materials accurately. Moreover, the integration of physics-based and data-driven approaches highlights the potential for enhanced scalability, but this requires the development of robust frameworks for data fusion and standardisation [17,230].

Cross-domain integration demands collaboration between civil engineers, software developers, and data scientists. However, inconsistent frameworks and data-sharing protocols often hinder seamless collaboration. Unified standards and enhanced communication across stakeholders are essential to facilitate the integration of DT systems into diverse domains such as civil infrastructure and smart cities [29]. Data security further complicates integration, as unauthorised access to sensitive information poses economic and privacy risks. Ensuring system integrity through user authentication and controlled data sharing is critical.

### 8.2. Real Time Performance

Real-time performance is critical for DT systems in SHM, where immediate decision-making is necessary for structural integrity and safety. However, achieving immediate decision-making is challenging due to substantial latency in data processing and synchronisation between physical and digital twins. The inability of current computational infrastructures to support high-frequency data updates highlights the need for more advanced telemetry systems and wireless communication technologies [14,33]. While frequent updates improve real-time accuracy, they impose significant demands on communication networks, requiring a careful balance between resource constraints and system performance. Simulation methods that require real-time updates face similar challenges. Finer mesh models, while improving accuracy, increase computational demands, underscoring the trade-off between accuracy and real-time feasibility [121].

Maintaining seamless monitoring between physical objects and their virtual twins presents further challenges, including high data traffic and potential transmission errors. Ensuring stable internet connections and minimising interference are crucial to sustaining real-time functionality. Additionally, setting accurate thresholds for warnings is complex, particularly for ageing structures where material deterioration affects performance parameters. Threshold determination must account for structural ageing to avoid false alarms or missed warnings. Inverse methods are often necessary to determine structural loads from sensor data. However, these methods are inherently ill-conditioned, limited to specific measurement points, and prone to inaccuracies. The linear nature of inverse model updating further complicates analysis, especially under non-linear structural behaviour during extreme events such as earthquakes [120,231].

### 8.3. Computational Cost vs. Accuracy

The balance between computational cost and accuracy is a critical consideration in DT development. High-fidelity modelling techniques like Finite Element Modelling (FEM) provide exceptional accuracy but demand significant computational resources, making them impractical for real-time applications in large-scale infrastructure. Simplified techniques, such as adaptive mesh refinement and model order reduction, offer potential solutions but remain underutilised in practice [17,19]. Sensor selection and placement play a pivotal role in managing the balance between computation cost and accuracy. For example, strain gauges are suitable for stress evaluation, while vibrometers are better suited for dynamic assessments. Selecting the right sensors ensures relevant data collection while minimising computational overhead.

The architecture of DT systems must also be designed to optimise computational effort and system capabilities. This architecture development involves tailoring physical, data acquisition, integration, and digital twin layers to manage data processing effectively. Similarly, selecting appropriate software platforms is crucial, as functionality, licencing fees, and resource demands vary widely. Machine learning models, such as decision trees and ensemble methods, provide opportunities to streamline computational processes while maintaining accuracy. These methods improve generalisation capabilities and reduce overfitting, making them particularly valuable for predictive tasks in SHM [78]. However, DT models require extensive training before deployment. Measured data often include errors due to device inaccuracies or environmental influences, complicating the training process [120].

### 8.4. Research Gaps and Challenges

The implementation of DT systems faces numerous barriers. Advanced methods like shape sensing and inverse FEM (iFEM) encounter high costs associated with sensor deployment and computational demands. Moreover, these methods are often confined to laboratory settings, limiting their application to real-world scenarios due to reliance on fragile and expensive sensors [38,40]. A significant challenge is predicting structural failures through comparative analysis of calculated and measured responses. This task requires high model fidelity and accurate real-time synchronisation, both of which remain underdeveloped in current systems [119].

While machine learning and artificial intelligence offer significant promises for enhancing SHM systems, their application remains limited. Existing research often focuses on traditional methods, neglecting ML’s potential to manage large, heterogeneous datasets and optimise real-time performance [42]. DT models must also integrate environmental factors such as ground motions and soil-structure interaction. Techniques like augmented reality and Foundation Input Motions (FIMs) are promising but require further exploration to ensure sensitivity to real-world conditions [120,121]. Additionally, the transformation of measurement data into virtual twins involves complex processes, including communication, validation, and synchronisation, each presenting unique challenges. The lack of digital data for ageing infrastructure presents another significant gap. Collecting new datasets for older structures is often resource-intensive, further complicating efforts to develop accurate DT systems.

## 9. Conclusions

DT, a digital replica of a physical structure, is a very useful tool in monitoring structural health in real time for the conditions’ assessment as the replacement for the conventional visual inspection and non-destructive test methods. While DTs have been used in various sectors, their development in civil infrastructure engineering is still very limited. DT can significantly help intelligent monitoring and maintenance of civil infrastructure. The designer of a DT must consider comprehensive architecture in determining the methods for obtaining and transferring the data in real time by using advanced IoT technology installed on an existing structure. Creating the digital model also requires the selection of appropriate software which can be effective in determining the boundary conditions that will have minimum differences in representing the physical world. The approach toward the modelling and the type of configuration is most effective for the selection of the data-driven method. The platform development, which will be used as the hub of the data exchanger and interpretation when obtaining data from sensors and feeding them into the model, is very important.

Lastly, the challenges that a DT designer confronts are also very critical and the designer must be able to resolve any challenges before shifting towards real-time DT development, as these can have impacts on the accuracy of the developed DT.

## Figures and Tables

**Table 1:** DT definitions in various industries over time.

Scholar	Definition of DT
Shafto, et al. [19]	A wholistic probabilistic simulation that efficiently utilises physical models, sensor updates, and more that duplicate the flying twin. The DT is reflective and includes multiple interdependent vehicle systems.
Glaessgen and Stargel [20]	An ultra-fidelity simulation that combines multiphysics and multiscale models with probabilistic methods to promptly reflect the state of its corresponding physical system, utilising historical data, real-time sensor inputs, and physical models.
Rosen, et al. [21]	Highly accurate models depict a process’s present state and behaviour and interact with real-world surroundings.
Gabor, et al. [22]	A sophisticated simulation that accurately depicts the asset’s overall behaviour by integrating previously separated models of various structural design aspects, enabled by increased computational power.
Schluse and Rossmann [23]	Virtual replacements of physical items composed of virtual representations and communication capabilities. These objects function as intelligent nodes within the Internet of Things and services.
Canedo [24]	A digital rendering of a tangible thing with emphasis on the object itself.
Eigner, et al. [25]	DTs exit throughout the entire lifecycle in the form of virtual models and can be subdivided into the phases “as-designed”, “as-built”, and “as-maintained”.
Jones, et al. [26]	A combination of physical and virtual entities, environments and processes should fulfil the listed characteristics and features.
Qi, et al. [27]	The 5-dimensional DT model capable of serving as a universal reference embodiment to coordinate with engineering applications across several sectors.
Attaran and Celik [28]	A complex simulation of the physical asset and a platform to enable future technologies such as speech capabilities, augmented reality, IoT, and artificial intelligence (AI) beyond the limitations of conventional civil engineering.

**Table 2:** Methodology of screening papers.

Searching Index	Specific Content
Database	Scopus and Google Scholar.
Article Type	Scientific/technical articles published in peer-reviewed journals and conferences.
Search Strings	“Digital Twin”, “Built environment”, “Civil infrastructure”, “Data acquisition”, “Health monitoring”, “Digital Twin instrumentation”, etc.
Search Period	From January 2005 to December 2023.
Screening Procedure	The relevance of the research topic is judged by the contents of the abstract, introduction, and conclusion of every paper.
Classification Scheme	The development of current DT transformation, DT-enabling technologies for data collection and transmission, DT health assessment, and DT-related project risks.

**Table 3:** Applications of different shape sensing methods.

Scholar	Method	Capabilities	Requirements
Davis, et al. [124]	Optimised trial functions and weights	Reconstruction of a simple static beam response from discrete strain measurements	To model more complex deformations, the approach requires a large number of trail functions and strain sensors
Jones, et al. [125]	Least-squares formulation	Shape sensing of a cantilevered plate and plate deflections were obtained with classical bending assumptions	Axial strain fitted with a cubic polynomial
Shkarayev, et al. [99], Shkarayev, et al. [126]	Two-step solution procedure; structural analysis of a plate/shell FE model, least-squares algorithm	Shape sensing of the plate and shell element	Reconstructs the loads first and then moved for displacements
Bogert, et al. [127]	Modal transformation method—a large number of natural vibration modes were used	Strain-displacement transformations	Computationally intensive eigenvalue analysis and a detailed description of the elastic and inertial material properties for high-fidelity FE models
Kim and Cho [128]	Classical beam equations, regression of experimental strain data	Continuous curvature function to evaluate beam deflection	Plates/shell structures
Mainçon [129]	Finite Element Formulation	Sensitivity analysis is included for truss structures	Prior knowledge of a subset of applied loading and material properties
Kang, et al. [130]	Vibration mode shapes	Reconstruction of a beam response due to dynamic excitations	The same number of mode shapes and strain sensors were required
Ko, et al. [131]	Approximating the beam curvature using piece-wise continuous polynomials	Sufficiently accurate for predicting deflection and less accurate for assessing the cross-sectional twist	Included the bending and torsion modes of deformation
Nishio, et al. [132]	Weighted least squares formulation	Reconstruct the deflection of a composite cantilevered plate	Weights were calculated for a given data acquisition apparatus, load case, and test article

**Table 4:** Modelling and interpretation techniques.

Scholar	Tested Physical Model and Purpose	Approach/Method
Haag and Anderl [161]	Beam bending experiment	CAD and stress analysis tools
Jayasinghe, et al. [42]	Real-time SHM of a port structure	FE modelling with artificial neural networks
Lu and Brilakis [118]	Modelling bridges to create a DT	A slicing-based object fitting method incorporating four types of labelled point cluster
Ye, et al. [17]	SHM of bridges	Physics-based (FEM) and data-driven (linear dynamic modelling)
Shim, et al. [162]	SHM of cable-supported bridges	FE modelling
Kaewunruen, et al. [98]	Risk-based maintenance planning of bridges under extreme weather	BIM
Dan, et al. [119]	DT of a bridge with measured traffic flow	Machine vision techniques and BrIM
Lin, et al. [62]	Collapse fragility assessment of a bridge	FE modelling
Febrianto, et al. [163]	SHM of bridges	Statistical FE modelling
Ghahari, et al. [120]	Post-earthquake damage diagnosis of bridges	Output only Bayesian model updating technique through an FE model incorporating soil-structure interaction effects and foundation input motions
Mirasoli, et al. [164]	Bridge structure	FE high-fidelity modelling
Adibfar and Costin [160]	DT for a prototype Bridge	BIM

**Table 5:** Recent applications of BDT.

Lifecycle Phase	DT Application	Technologies	Scholar
Planning and Design	Design of a thermal system that is blended with a light-weight roof structure	High-resolution models	[216]
	A new generation parametric system, Packhunt.io was presented along with two real-world cases	BIM, Extended reality, visual programming	[217]
Construction	Automated construction progress monitoring system	BIM, Extended Reality Technologies	[218]
	Advanced project management framework for construction operations	BIM, Data Mining (DM) techniques	[219]
	Suggested a method to develop a DT for a building facade	Python coding	[215]
Operation and Maintenance	Dynamic DT demonstrator for a smart building as a proof of concept	BIM, WSN	[220]
	DT system architecture designed at both the building and city levels for the West Cambridge campus	BIM, Point Cloud Modal Generation, Anomaly Detection, Data Integration and Synchronisation	[220,221]
	Software reference architecture for creating and managing Smart Building DT	Sensor Data Management, Smart Building, BIM Ontology, IoT, and DT	[222]
	A city-scale DT–enabled urban energy management platform with benchmarking	Building Energy Benchmarking, Smart City	[223]
	An architecture design of transportation system DT of the smart city involving Dig Data and Bayesian Network Structural Learning Algorithm	Smart City, BIM, ITS, Multimedia Big Data, Bayesian Network Structural Learning Algorithm	[224]
